# Effects of Long-Term Airport Noise Exposure on Inflammation and Intestinal Flora and Their Metabolites in Mice

**DOI:** 10.3390/metabo15040251

**Published:** 2025-04-05

**Authors:** Jian Yang, Longwei Wei, Yuan Xia, Junyi Wang, Yan Bai, Yun Xia

**Affiliations:** School of Public Health, Guangdong Pharmaceutical University, Guangzhou 510006, China; 2112241062@gdpu.edu.cn (J.Y.); 2112415045@stu.gdpu.edu.cn (L.W.); xiayuan@gdpu.edu.cn (Y.X.); wangjunyi@gdpu.edu.cn (J.W.)

**Keywords:** airport noise, cardiovascular disease, vascular inflammation, microbiota, short-chain fatty acids

## Abstract

**Background**: The World Health Organization has indicated that airport noise is strongly associated with cardiovascular disease, with vascular inflammation identified as the primary mechanism. Therefore, long-term exposure to airport noise is considered far more harmful than other types of noise. However, there remains a lack of research into the mechanisms underlying long-term exposure to airport noise and harm to the human body. **Methods**: A mouse model was established and exposed to airport noise at a maximum sound pressure level of 95 dB(A) and an equivalent continuous sound pressure level of 72 dB(A) for 12 h per day over a period of 100 days. Quantitative polymerase chain reaction (qPCR) was used to detect the mRNA expression levels of pro-inflammatory and anti-inflammatory factors. Enzyme-linked immunosorbent assay (ELISA) was used to detect LPS, LTA, TMA, and TMAO levels. Intestinal flora composition was analyzed by 16S rDNA sequencing, and targeted metabolomics was employed to determine the levels of serum short-chain fatty acids. **Results**: Long-term airport noise exposure significantly increased systolic blood pressure, diastolic blood pressure, and mean blood pressure (*p* < 0.05); significantly increased the mRNA expression levels of oxidative stress parameters (nuclear matrix protein 2, 3-nitrotyrosine, and monocyte chemoattractant protein-1) (*p* < 0.05); significantly increased pro-inflammatory factors (interleukin 6 and tumor necrosis factor alpha) (*p* < 0.05); significantly decreased the mRNA expression level of anti-inflammatory factor interleukin 10 (*p* < 0.05); and significantly increased the content of LPS and LTA (*p* < 0.05). The composition of the main flora in the intestinal tract was structurally disordered, and there were significant differences between the noise-exposed and control groups at the levels of the phylum, family, and genus of bacteria. β-diversity of the principal component analysis diagrams was clearly distinguished. Compared with those of the control group, TMA-producing bacteria and levels of TMA and TMAO were significantly reduced, and the serum ethanoic acid and propanoic acid levels of the noise-exposed group were significantly decreased (*p* < 0.05). **Conclusions**: Long-term airport noise exposure causes significant elevation of blood pressure and structural disruption in the composition of the intestinal flora in mice, leading to elevated levels of oxidative stress and inflammation, resulting in metabolic disorders that lead to significant changes in the production of metabolites.

## 1. Introduction

The rapid development of urban transport has brought both convenience and prosperity, but also a series of environmental problems, especially noise pollution [1,2,3]. Existing studies have found that, among all traffic noise sources, aircraft noise is regarded as the most problematic [4]. Its primary mechanism involves eliciting excitation of the autonomic nervous system, such as by increasing heart rate and blood pressure [4]. Aircraft noise has been reported to be more offensive than other traffic noise at the same noise level [4]. Airport noise shares some common characteristics with other types of noise, such as sensitivity, localization, and temporality [5]. At the same time, airport noise also has some unique features, including predominantly low-frequency noise, which is much more hazardous than other types of noise because of its high diffractive power, reduced energy attenuation, and imperceptibility [6,7]. Moreover, due to the acceleration of urbanization and globalization, as well as the growing proximity between urban settlements and airports, airport noise is spreading closer to human residences. The negative health effects of airport noise are becoming increasingly prominent. In a recent study, aircraft noise was associated with increased blood pressure, endothelial dysfunction, and oxidative stress [8]. A meta-analysis that included 16,784 participants indicated that aircraft noise exposure leads to an increased incidence of hypertension in people living near airports [9].

Existing research on hypertension has focused on vascular structure and central neuromodulation, but the gut microbiota have not received sufficient attention. Structural disorders of the intestinal flora caused by unhealthy dietary habits, adverse environmental factors, and intestinal infections can alter the type and number of intestinal microorganisms, which can lead to inflammation and metabolic disorders and exacerbate intestinal flora dysbiosis. A growing number of studies have shown that imbalances in the intestinal microbiota play an integral role in the development and progression of hypertension [10] and that hypertension significantly affects the structure of the intestinal microbiota [11,12]. A study of a rat model of spontaneous hypertension and long-term angiotensin II (Ang II) injections revealed a significant decrease in the abundance, diversity, and homogeneity of the gut microbiota and a significant decrease in the proportion of gut microbes. An increase in the phylum Fusobacterium *Firmicutes/Bacteroidetes* (*F/B*) revealed an imbalance in the gut microbiota of hypertensive animals [13]. In another population-based study, the gut microbiota richness, diversity, and gene counts were significantly lower than in participants with prehypertension and hypertension compared with healthy participants [10]. The results of a cohort study examining the relationship between intestinal microbiota and hypertension indicated that the diversity of the gut microbiota was negatively correlated with hypertension [14].

The structure and composition of intestinal microbiota have been found to differ significantly among hypertensive patients with different cardiovascular risk levels, and some intestinal microorganisms have been strongly correlated with the severity of hypertension, indicating that the intestinal microbiota may be involved in the onset and development of hypertension [15]. These findings indicate that changes in the structure and composition of intestinal microbiota are important factors in the development and progression of hypertension. In one study, noise exposure patterns were reported to alter the percentage of *Proteus species* and *Actinobacillus* in the gut, increasing the abundance of *Roseburia* [16]. At the same time, the abundance of the probiotic *Faecalibacterium prausnitzi* in the cecum decreased, which ultimately led to intestinal inflammation and disturbances in glucose metabolism [16]. In addition to directly altering the intestinal microbiota, noise exposure caused several of their metabolites to enter the body’s circulation, causing disease.

Although current research is beginning to emphasize the role that gut flora and their metabolites play in the inflammatory response and their involvement in the management of hypertension, the exact mechanisms underlying these responses are not clearly understood. Trimethylamine (TMA) and trimethylamine oxide (TMAO) are metabolites of the intestinal flora and have a multifaceted impact on human health. Physiological concentrations of TMA disrupt tight junctions and increase the permeability of the blood–brain barrier (BBB) [17]. Although the specific roles of TMAO in the body are not yet fully defined, studies in recent years have correlated it with a variety of chronic diseases (e.g., cardiovascular disease, diabetes, etc.). Elevated circulating levels of TMAO may independently predict the risk of major adverse cardiac events [18,19,20]. In addition to changes in the intestinal microbiota, some of their metabolites—such as lower carboxylic acids, which are primarily composed of ethanoic acid and propanoic acid—enter the circulation and contribute to the development of disease.

Airport noise is different from general noise, being characterized by low-frequency noise, strong diffraction ability, and reduced energy attenuation. Unnoticeable, long-term exposure to airport noise is significantly more dangerous to the human body than other noise. Therefore, we established a mouse model exposed to airport noise at a maximum sound pressure level of 95 dB(A) and an equivalent continuous sound pressure level of 72 dB(A) for 12 h per day over 100 days. We investigated the effects of inflammation and intestinal flora and their metabolites on mice to examine the mechanism of intestinal flora and their metabolites in the inflammatory reaction to airport noise.

## 2. Materials and Methods

### 2.1. Animal Grouping and Airport Noise Exposure

Thirty 6-week-old male C57BL/6J mice were randomized into a control group and a noise-exposed group of 15 mice in each group. The noise-exposed group was repeatedly exposed to an unstable aircraft noise pattern lasting 5 min with a maximum sound pressure level of 95 dB and an equivalent continuous sound pressure level of 72 dB, delivered through a loudspeaker for 12 h per day, from 8:00 to 20:00, over a period of 100 days. The control group was housed in an environment with background noise levels below 50 dB. The animals were kept at a temperature of 23 ± 2 °C, a humidity of 50–60%, a light–dark cycle of 12 h, and had free access to food and water. All animal experiments were conducted according to the Chinese guidelines for animal protection and welfare.

### 2.2. Noninvasive Blood Pressure Test

Measurements were taken at 7-day intervals from the first day of noise exposure using a noninvasive blood pressure measurement system. Three training sessions were conducted before the measurements to acclimate the animals to the environment before the official measurements. Awake animals were allowed free access to the restraint tubes and rested for 15 min in the restraint tubes at a constant temperature (30 °C). Fifteen consecutive measurements were taken per animal, with the first 5 measurements discarded as part of the acclimatization cycle. The average of the remaining 10 measurements was used for calculations.

### 2.3. Effect of Noise Exposure on Inflammatory Factor Levels

The expression of C-reactive protein (CRP), vascular cell adhesion molecule 1 (VCAM-1), interleukin 6 (IL-6), tumor necrosis factor alpha (TNF-α), NOX-2, 3-nitrotyrosine (3-NT), monocyte chemoattractant protein-1 (MCP-1), and IL-10 genes in the mouse colon was detected by quantitative polymerase chain reaction (qPCR). RNA was extracted by adding TRIzol to the samples, followed by adding chloroform and performing centrifugation. Isolated RNA was precipitated with isopropanol, rinsed with ethanol, and then detected by qPCR performed under the following conditions: incubation at 95 °C for 30 s, followed by 40 cycles of 95 °C for 15 s, 55 °C for 20 s, and 72 °C for 20 s.

### 2.4. Targeted Metabolomics Analysis

Samples were obtained: (1) After thawing the serums, vortex the serums for 1 min to mix (all vortexing operations were performed with the vortexer frequency set to maximum). (2) Take 50 μL of the serum and add it to the corresponding 1.5 mL centrifuge tube, add 100 μL of 0.5% phosphoric acid solution, and vortex for 3 min. (3) Add 150 μL of MTBE solvent containing internal standards (the internal standards in the MTBE solution were acetic acid-d3 200 μg/mL, propionic acid-d5 200 μg/mL, butyric acid-d7 200 μg/mL, 2-methylpentanoic acid 80 μg/mL, isooctanoic acid-d15 40 μg/mL), vortex for 3 min, and sonicate for 5 min in an ice bath; then centrifuge the sample for 10 min at 12,000 r/min and 4 °C. (4) After centrifugation, pipette 90 μL of the upper layer of the supernatant into an injection bottle lined with a glass tube, and store in the refrigerator at −20 °C for GC-MS/MS analysis. For the analysis of lower carboxylic acids, the method was as follows: (1) sample pretreatment: under acidic conditions (pH 2–3, hydrochloric acid adjustment), combine with ethyl acetate serum for extraction of carboxylic acids; (2) add acetonitrile to precipitate impurities, centrifuge, and remove the supernatant; (3) Silylation: after drying, add 50–100 μL of BSTFA and pyridine, then heat at 70 °C for 30 min to produce trimethyl silane (TMS) derivatives.

Samples of plasma or serum were thawed and vortexed for 1 min prior to analysis. 50 μL of samples were added to a 1.5 mL EP tube and 100 μL of phosphoric acid (0.5% *v*/*v*) solution was added to the EP tube. The mixture was vortexed for 3 min. 150 μL MTBE (containing internal standards) solution was added. The mixture was vortexed for 3 min and ultrasonicated for 5 min. After that, the mixture was centrifuged at 12,000 r/min for 10 min at a temperature of 4 °C. The supernatant was collected and used for GC-MS/MS analysis.

### 2.5. GC-MS/MSanalysis

An Agilent 7890B gas chromatograph coupled to a 7000D mass spectrometer with a DB-FFAP column (30 m length × 0.25 mm i.d. × 0.25 μm film thickness, J&W Scientific, Folsom, CA, USA) was employed for GC-MS/MS analysis of SCFAs. Helium was used as the carrier gas, at a flow rate of 1.2 mL/min (the split parameters were 5:1, and the gas consumption for the split was 6 mL/min). Injection was conducted in the split mode and the injection volume was 2 μL. The oven temperature was held at 90 °C for 1 min, increased to 100 °C at a rate of 25 °C/min, then to 150 °C at a rate of 20 °C/min, held for 0.6 min, and finally increased to 200 °C at a rate of 25 °C/min, and held for 0.5 min after running for 3 min. All samples were analyzed in multiple reaction monitoring mode. The injector inlet and transfer line temperatures were 200 °C and 230 °C, respectively. After obtaining the mass spectrometry analysis data for the different samples, the chromatographic peaks of all targets were integrated and quantitatively analyzed using standard curves.

### 2.6. Effect of Noise Exposure on LPS, LTA, TMA, and TMAO Levels

The levels of lipopolysaccharide (LPS), lipoteichoic acid (LTA), trimethylamine (TMA), and trimethylamine oxide (TMAO) in mouse serum were determined by enzyme-linked immunosorbent assay (ELISA), and the specific procedures were performed according to the kit’s instructions.

### 2.7. Sequencing and Analysis of 16s rDNA of Intestinal Flora

Approximately 200 mg of cecum content was collected from the mice after dissection. After genomic DNA extraction was performed according to the instructions of the DNA extraction kit, the integrity and purity of the DNA were assessed using 1% agarose gel electrophoresis, and the purity and concentration of the DNA were determined using NanoDropOne (Thermo Scientific, New York, NY, USA). The obtained DNA was used as a template, the bacterial 16S r DNA V3-V4 variable region was used as a target, and the primers with barcodes were used for amplification (upstream primer 338F: ACTCCTACGGGGAGGCAGCA; downstream primer 806R: GGACTACHVGGGTWTCTAAT). Up-sequencing was performed using the high-throughput sequencing platform Hiseq. The raw image data files obtained from sequencing were converted into the original sequenced sequences by base recognition analysis and then analyzed.

### 2.8. Preparation of Graphics and Statistical Analysis

The results of quantitative analysis were expressed as the mean ± standard deviation. Graphics were processed and statistical analysis was performed using SPSS 16.0 software (IBM, New York, NY, USA). Significant differences were distinguished at a probability (*p*) value of < 0.05.

## 3. Results

### 3.1. Effect of Chronic Airport Noise on Blood Pressure in Mice

**Figure 1:**
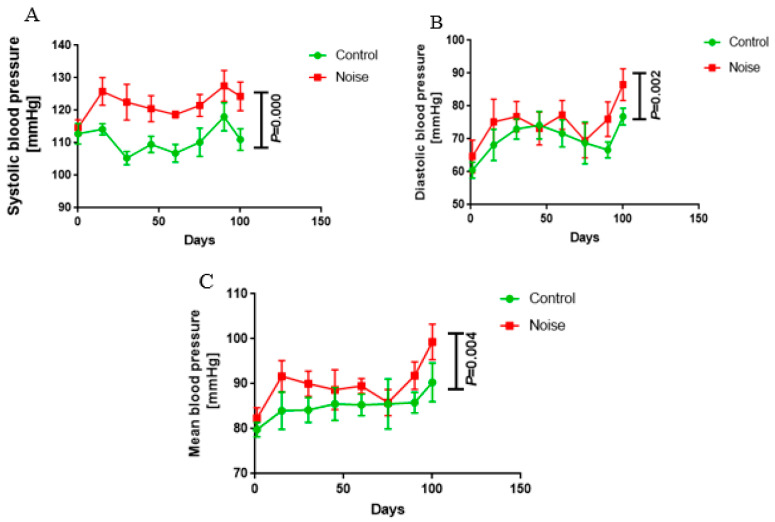
Changes in systolic (**A**), diastolic (**B**), and mean blood pressure (**C**) during exposure to chronic airport noise.

After 100 days of airport noise exposure, the mice in the noise-exposed group had a systolic blood pressure of 124 ± 4.46 mmHg, a diastolic blood pressure of 86.50 ± 4.85 mmHg, and a mean blood pressure of 99.33 ± 3.98 mmHg, which were significantly higher than those of the control group (systolic blood pressure of 111.0 ± 3.35 mmHg, diastolic blood pressure of 76.83 ± 2.56 mmHg, and mean blood pressure of 90.33 ± 4.32 mmHg; *p <* 0.05) (Figure 1A–C). These results indicate that airport noise exposure causes elevated blood pressure in mice.

### 3.2. Changes in Oxidative Stress, Inflammatory Markers, and LPS and LTA Levels in Mice After Chronic Airport Noise Exposure

**Figure 2:**
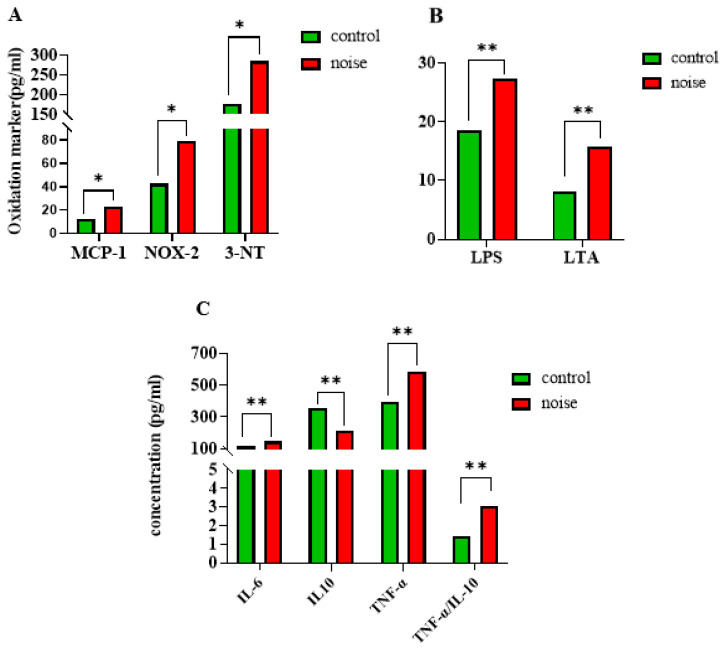
(**A**) Changes in serum oxidative stress markers in mice after exposure to chronic airport noise. (**B**) Changes in serum LPS and LTA in mice after exposure to chronic airport noise. (**C**) Changes in inflammatory and anti-inflammatory factors in cardiac tissues of mice after exposure to chronic airport noise. * *p* < 0.05, ** *p* < 0.01.

After 100 days of noise exposure, levels of MCP-1 (19.26 ± 2.56 noise-exposed group vs. 14.37 ± 2.73 control group), NOX-2 (69.83 ± 10.47 noise-exposed group vs. 55.96 ± 10.98 control group), and 3-NT (257.08 ± 34.08 noise-exposed group vs. 218.07 ± 31.01 control group) were significantly elevated in the noise-exposed group compared with the control group (*p <* 0.05) (Figure 2A). Serum levels of LPS and LTA were significantly higher in the noise-exposed group compared with the control group (*p <* 0.05) (Figure 2B). Compared with the control group, the mRNA expression levels of the anti-inflammatory factor IL-10 were significantly lower and the mRNA expression levels of the pro-inflammatory factors IL-6 and TNF-α were significantly higher in the noise-exposed group (*p <* 0.05 for all measurements) (Figure 2C).

### 3.3. Effects of Chronic Airport Noise Exposure on Colon Tissue Structure Changes in Mice

**Figure 3:**
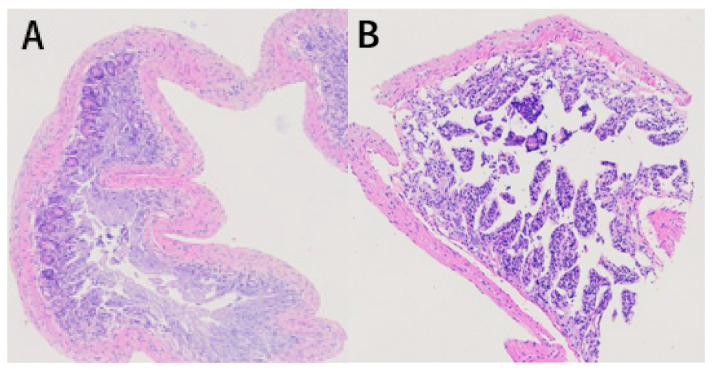
Hematoxylin and eosin staining results of colon tissue of mice exposed to chronic airport noise. (**A**) Colon tissue of the control group. (**B**) Colon tissue of the noise-exposure group.

In the control group, all the structures of the colon were arranged in an orderly manner; no damage was found in the intestinal epithelium, and the structure of the colonic crypts and mucosa was relatively complete (Figure 3A). In the noise-exposed group, colonic fracture and disordered arrangement, recess fracture and distortion, slightly swollen colon epithelial mucosa, reduced goblet cells, obvious inflammatory infiltration, irregular arrangement of colon recess structure, and obvious damage were observed (Figure 3B).

### 3.4. Effects of Chronic Airport Noise Exposure on Intestinal Flora Structure Changes in Mice

**Figure 4:**
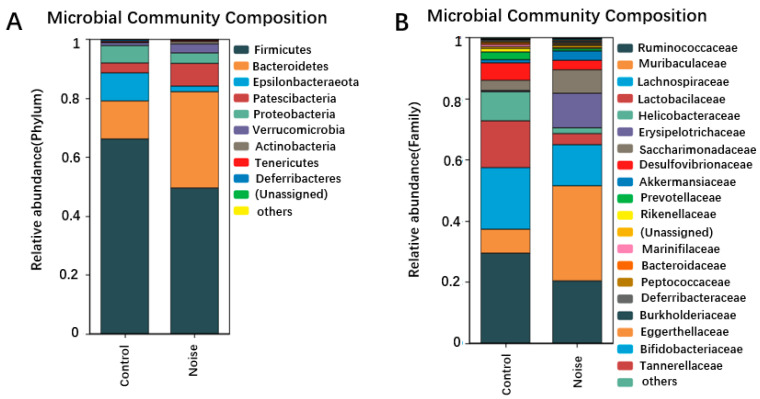

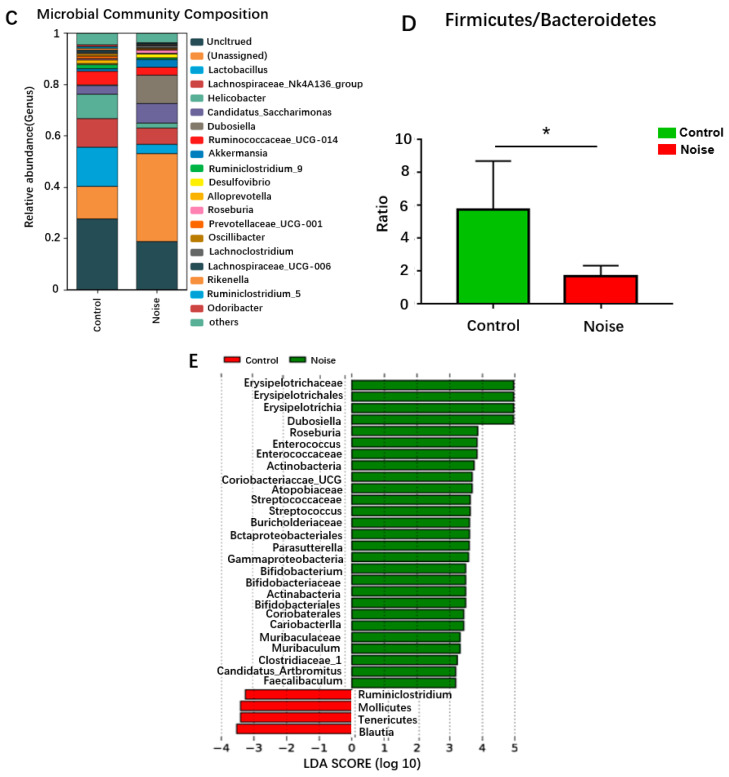
Changes in the composition of intestinal flora at the phylum (**A**), family (**B**), and genus (**C**) levels before and after noise exposure. (**D**) Changes in the Firmicutes/Bacteroidetes ratio before and after noise exposure. (**E**) LEfSe analysis of differential clusters between taxa from phylum to genus. * *p* < 0.05.

Our research group previously assessed the structure of mouse intestinal flora by analyzing DNA extracted from cecum content by 16S sequencing. As shown in Figure 4A, at the gate level, nine dominant bacteria were identified in the control group and noise-exposure group (Figure 4A). The intestinal flora of mice were dominated by *Firmicutes* (57.9%) and *Bacteroidetes* (22.8%), followed by *Epsilonbacteraeota* (5.7%), *Patescibacteria* (5.5%), Proteobacteria (4.7%), and *Verrucomicrobia* (2%). Three phyla showed a relative abundance <0.5%: *Actinobacteria* (0.039%), *Tenericutes* (0.0156%), and *Deferribacteres* (0.0111%). The number of Mycobacterium simulans in the phylum Actinomycetota was significantly increased in the noise-exposed group compared with the control group *(p <* 0.05).

Changes in the intestinal bacterial compositions were also observed at the family level (Figure 4B). The relative abundance of *Muribaculaceae* and *Erysipelotrichaceae* was significantly *(p <* 0.05) increased in the noise-exposed group compared with the control group. When the microbiome composition of the different taxa was compared at the genus level (Figure 4C), unassigned bacteria were found to significantly increase and *Lachnospiraceae_UCG-006* to significantly decrease in the noise-exposed group compared with the control group *(p <* 0.05). The proportion of thick-walled phylum/Anthrobacterium was significantly reduced in the noise-exposed group correlated with the control group (*p* < 0.05) (Figure 4D).

Simpson’s diversity index analysis demonstrated no significant difference in alpha diversity between the two groups, indicating no differences in the species, richness, or evenness of the bacterial communities between the two groups. Using weighted Unifrac distances to assess the overall differences in intestinal microbiota between the noise-exposed and control groups, principal coordinates 1 and 2 (PCoA1 and PCoA2) were found to explain 60.6% and 22.5% of the variations in Bray–Curits differences, respectively. The noise-exposed group was clustered differently than the control group, and although the two clusters were not completely separated, they showed some degree of differentiation. The microbial communities of the noise-exposed group did not significantly differ (*p >* 0.05) from those of the control group in the alpha diversity indexes, including the richness, chao1, reads, and Simpson’s indexes.

Linear discriminant analysis effect size (LEfSe) analysis is a way of discovering and interpreting high-dimensional biomarkers (e.g., genes, pathways, and taxonomic units) that can be used to make comparisons of two or more subgroups. Emphasizing statistical significance and biological relevance, LEfSe analysis can identify biomarkers that differentiate one group from another and features of varying abundance, as well as associated categories. The linear discriminant analysis (LDA) bar graph represents the magnitude of the influence of a bacterium in a group. The LDA was set at 2.0 in the subject experiment, meaning that an LDA > 2.0 indicated a significantly different species. The analysis showed that *Ruminiclostridium*, *Blautia*, soft-walled bacteria, and *Mollicutes* of thick-walled bacteria were enriched in the control group, whereas *Actinobacteria*, the *Coriobacteria* family of Actinobacteria, the *Bacteroidia* family of Mycobacteria and their genera, *Bacilli*, thick-walled bacteria, Clostridia, Erysobacteria and their genera, *Clostridium*, *Erysipelotrichia* and their genera, and *Gammaproteobacteria* and their genera of Ascomycetes were enriched in the noise-exposed group (Figure 4E).

### 3.5. Effects of Chronic Airport Noise Exposure on Levels of Trimethylamine and Trimethylamine Oxide

**Figure 5:**
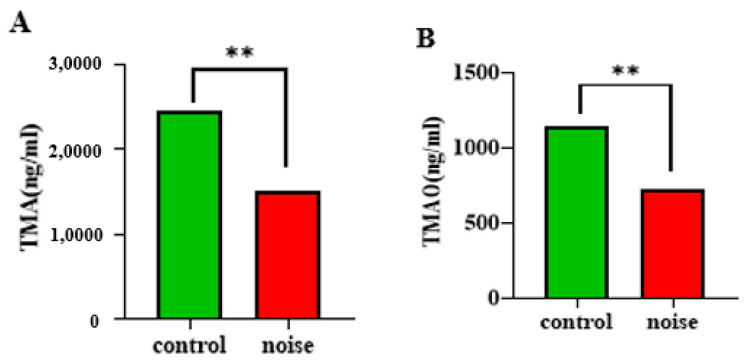
Changes in TMA (**A**) and TMAO (**B**) levels in mice before and after chronic airport noise exposure. ** *p* < 0.01.

Trimethylamine (TMA) and trimethylamine oxide (TMAO) are important products of intestinal microbial metabolism, and they are involved in relevant physiological responses when they enter the human circulation. TMAO is strongly correlated with the development and progression of hypertension. Noise exposure resulted in significantly lower serum levels of TMA and TMAO (*p <* 0.05) (Figure 5A,B). Low levels of TMA and TMAO lead to disturbed energy metabolism, which in turn leads to significant changes in the levels of other metabolites.

### 3.6. Effect of Chronic Airport Noise Exposure on Serum Lower Carboxylic Acid Levels

**Figure 6:**
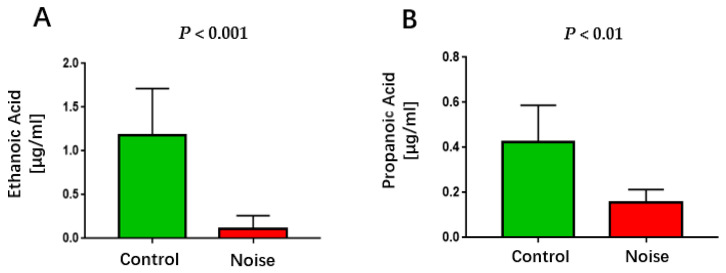
Changes in serum ethanoic acid (**A**) and propanoic acid (**B**) levels after chronic airport noise exposure.

Chronic exposure to airport noise resulted in a significant reduction in serum levels of ethanoic acid and propanoic acid (Figure 6) (*p* < 0.01).

### 3.7. Correlation Among Microbial Composition, Blood Pressure, and Intestinal Metabolites in Mice

**Figure 7:**
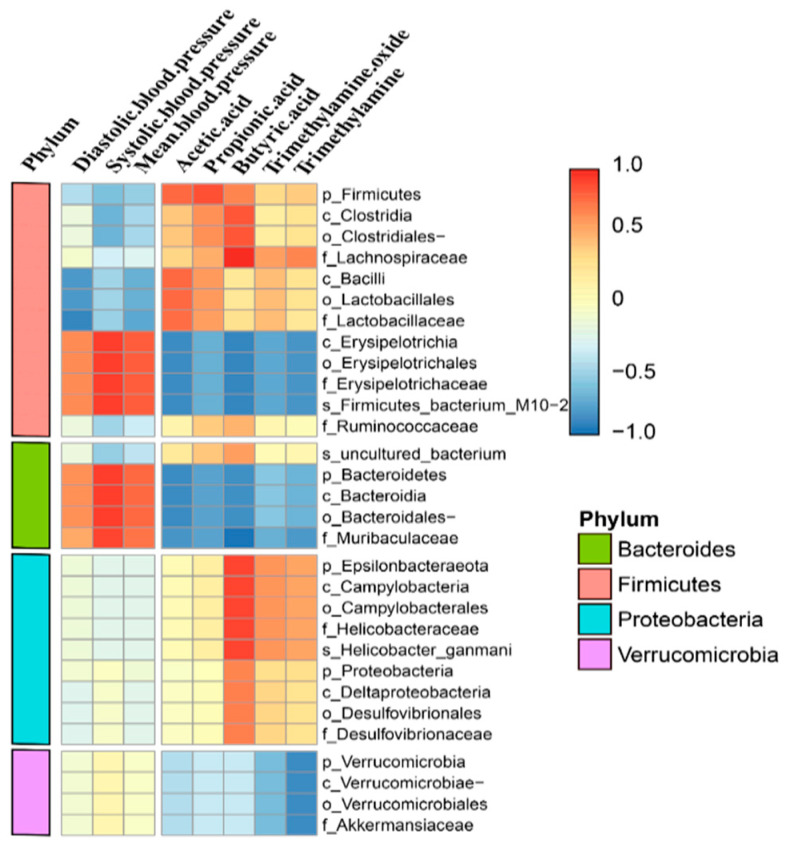
Correlation analysis of biological structures with blood pressure and serum metabolites.

To further explore the correlation between blood pressure and intestinal flora, we calculated the Pearson correlation coefficient to generate a correlation matrix according to different levels of bacteria for correlation analysis (Figure 7). We identified seven microbiomes that were significantly associated with blood pressure. Among them, Erysipelas and Bacteroides were positively correlated with blood pressure, while *Clostridium*, *Bacillus*, *Lactobacillus*, and *Lactobacillaceae* were negatively correlated with blood pressure. For trimethylamine and trimethylamine oxides, we identified microflora negatively associated with these variants, including *Erysipelotrichia*, *Bacteroidetes*, *Muribaculaceae*, and *Verrucomicrobia*.

## 4. Discussion

Previous studies have demonstrated that traffic noise exposure is a commonly occurring environmental health hazard that causes metabolic disorders [16] and an increased risk of cardiovascular disease (CVD) [21,22]. As urbanization has continued to develop worldwide, traffic noise pollution has also increased. The development of transportation, including airports, railroads, and road traffic, has led to increased environmental noise exposure. Nevertheless, studies investigating the effects of long-term airport noise exposure on the cardiovascular system and its relationship with intestinal flora remain scarce, and the mechanisms underlying the effects of noise on the body remain unclear. Moreover, most animal studies have used high-intensity white noise to examine the effect of noise on the body [23,24,25].

Therefore, we established a mouse model exposed to airport noise conditions with a 95 dB(A) maximum sound pressure and a 72 dB(A) equivalent continuous sound pressure level, for 12 h per day over a period of 100 days. This model was used to observe the effects of long-term airport noise on inflammation and the structure of intestinal flora and their metabolites in mice. We found that long-term airport noise exposure in mice caused a significant increase in blood pressure, enhanced levels of oxidative stress and inflammatory responses, and caused structural disturbances in the composition of the intestinal microflora and alterations in the levels of their metabolites. Based on the results we obtained in this study, we can conclude that chronic airport noise exposure leads to a significant increase in systolic, diastolic, and mean blood pressure in mice (*p* < 0.05). Specifically, we observed that the mRNA expression levels of inflammatory factors such as L-6 and TNF-α significantly increased (*p* < 0.05); serum levels of oxidative stress markers such as MCP-1, 3-NT, and NOX-2 significantly increased (*p* < 0.05); and the mRNA expression level of the anti-inflammatory factor IL-10 significantly decreased (*p* < 0.05). These findings are consistent with previous studies on the impact of long-term exposure to aircraft noise [26,27,28].

Aircraft noise has been a persistent problem in developed countries and an escalating problem in developing countries, where it is increasingly damaging to the health of residential communities. There is convincing evidence that noise exposure is a cardiovascular risk factor. Several large epidemiological studies have demonstrated that traffic noise is correlated with an increased risk of CVD. Among the types of traffic noise, airport noise is particularly correlated with an increased risk of CVD, as it induces hypertension, endothelial dysfunction, and elevated levels of vascular oxidative stress and inflammation [8,29,30,31,32]. According to a systematic analysis of the 2018 World Health Organization Guidelines on Ambient Noise in the European Region, exposure to road traffic noise increases the risk of ischemic heart disease (IHD) [33]. The pooled relative risk of IHD was 1.08% (95% confidence interval 1.01–1.115) for each 10 dB(A) rise in traffic noise exposure, with significant adverse health effects at noise levels >50 dB(A). Even more striking are the recent data from Saucy et al. [5]: among nearly 25,000 cases of CVD in a Swiss national cohort near Zurich International Airport, exposure to noise in the two hours preceding nocturnal death was significantly correlated with all-cause mortality.

The intestinal microbiota play an integral role in sustaining blood pressure stability. Alterations in the structure of the intestinal microbiota are strongly correlated with the development and progression of hypertension [34]. Experiments using fecal microbiota transplanted into sterile animals have indicated that the intestinal microbiota not only correlate with elevated blood pressure but can also directly elevate it [10,35]. This elevation may involve the activity of intestinal microbial metabolites, such as SCFAs and lower carboxylic acids, which participate in related reactions in the body. In this study, examination of intestinal microbiota and their metabolites indicated that long-term airport noise exposure caused alterations in the compositional structure of the intestinal microbiota at the level of the phylum, with a decrease in the abundance of the predominant phylum; a decrease in the abundance of the thick-walled phylum; an increase in the abundance of the anaplastic phylum; and a significant decrease in the ratio of thick-walled phylum to anaplastic phylum (*F/B*), an important parameter reflecting the imbalance of the intestinal microbiota. The imbalanced state of intestinal microbiota, especially as reflected in the *F/B* ratio, is correlated with changes in blood pressure. A previous study employing a rat model of hypertension noted that *F/B* was significantly increased [36]. At the genus level, the noise-exposed group showed an increased abundance of *Candidatus_Saccharimonas*, a conditionally pathogenic bacterium that exists throughout the gastrointestinal (GI) tract and is correlated with inflammatory mucosal diseases in humans, demonstrating its possible involvement in inflammatory reactions [37].

LEfSe analysis identified bacteria that differed significantly between the noise-exposed and control groups. *Erysipelotrichaceae,* whose abundance was elevated in the noise-exposed group, and which belongs to the phylum of thick-walled bacteria, has been correlated with GI inflammation-related disorders. Increased abundance of *Erysipelotrichaceae* has also been observed in the intestinal tract of patients with colorectal cancer [38]. Moreover, the corresponding relative abundance of *Erysipelotrichaceae* has been positively correlated with TNF-α concentration and colonic intestinal barrier integrity [39,40].

We observed that the serum level of LPS in the noise-exposed group was elevated, indicating that the intestinal barrier had been damaged to a certain extent, which may have manifested in severe damage to the morphological structure of the colon. This damage was confirmed by the results of our hematoxylin and eosin (HE) staining experiments. These results are consistent with the increased abundance of *Erysipelotrichaceae* in the noise-exposed group. We therefore hypothesize that *Erysipelotrichaceae* causes LPS to enter the bloodstream, leading to elevated levels of inflammation throughout the body through damage to the intestinal barrier and elevated levels of TNF-α [41].

We also found that the genus *Dubosiella* increased in abundance in the noise-exposed group. We analyzed functional correlations among microorganisms, blood pressure, and metabolites based on different levels of bacteria using Pearson correlation coefficients to generate correlation proofs. The results showed that *Dubosiella* spp. were significantly and negatively correlated with ethanoic acid and butanoic acid, while *Erysipelotrichia* in the Firmicutes phylum of thick-walled bacteria, including its orders and families, was positively correlated with blood pressure and negatively correlated with TMAO, monoamine oxidase (MAO), and SCFAs. Moreover, TMAO and MAO were strongly positively correlated with systolic blood pressure and strongly negatively correlated with TMAO, MAO, ethanoic acid, and butanoic acid. The family *Muribaculaceae* showed a strongly positive correlation with systolic blood pressure and a strongly negative correlation with SCFAs. These results are consistent with those of previous studies.

TMA is primarily produced by gut microorganisms that break down nutrients such as choline and carnitine [42], and is absorbed into the circulation, where it is oxidized to TMAO by the hepatic rate-limiting enzyme heparin monooxygenase 3 (FMO 3) [43]. There have been many studies demonstrating that high levels of TMA and TMAO are correlated with an increased risk of cardiovascular and metabolic diseases such as atherosclerosis, heart failure, and other disorders, possibly through effects on cholesterol metabolism and inflammatory responses [44,45,46,47]. However, in the present study, the levels of TMA and TMAO, which play important roles in cellular osmoregulation and help cells to maintain stability in high osmotic pressure environments, were significantly reduced in noise-exposed mice compared to the control group [44]. Therefore, low levels of TMAO may impair cellular osmoregulation, particularly in organs such as the kidney and liver. In addition, significantly lower levels of Clostridium were observed in the noise-exposed group compared to the control group. Consistent with the above results, one of the functions of Clostridium is to convert choline into TMA; these findings suggest that the intestinal flora are insufficiently diverse or functionally impaired, and that dysbiosis of the intestinal flora may further affect immune modulation, nutrient absorption, and metabolic health [48]. Indirectly, these findings suggest that the TMAO metabolic pathway may have bidirectional effects on health. Low levels of TMA may indirectly reflect abnormalities in carnitine and choline metabolism, and studies have pointed out that choline deficiency can lead to fatty liver and neurological damage, whereas carnitine deficiency may affect energy metabolism, and thus low levels of TMA may indirectly increase other metabolic risks, which may in turn affect hepatic, muscular, and neurological function [49].

Primarily composed of acetic, propionic, and butanoic acids, lower carboxylic acids are critical metabolites of the intestinal flora that play an integral role in energy metabolism and homeostasis of the intestinal environment. Several studies have demonstrated that lower carboxylic acids can alleviate inflammation [50,51,52,53,54,55]. Although several clinical studies have demonstrated that fecal lower carboxylic acid levels are significantly higher in patients with hypertension than in normal controls, serum lower carboxylic acid levels have been negatively correlated with blood pressure [56,57]. The most abundant lower carboxylic acid in the colon is ethanoic acid, which is involved in the anti-inflammatory reaction in the body by participating in the concentration of neurotransmitters, glutamate, and glutamine secreted by the hypothalamus [58]. In vivo experiments have shown that the supplementation of drinking water with butyrate or acetate prevents blood pressure elevation in spontaneously hypertensive rats (SHRs) [59]. Propionate reduces TNF-α and nitric oxide synthase (NOS) expression in LPS-induced haploidentical monocytes [60]. In this study, we observed that long-term airport noise exposure caused a significant decrease in serum levels of ethanoic acid and propanoic acid (*p <* 0.05), as well as a significant increase in blood pressure, findings that accorded with those of previous studies.

The overall permeability of the gut is low when gut barrier function is normal, a characteristic that effectively inhibits the entry of intestinal pathogens and exogenous toxins into the body and reduces the likelihood of an inflammatory response in the intestinal vasculature. When the barrier function is compromised, the overall permeability of the gut increases. Increased permeability permits toxic substances to cross the barrier and enter the bloodstream, causing the overproduction of a variety of pro-inflammatory factors, thereby exacerbating damage to the barrier function. At the same time, levels of pathogenic bacteria and LPS in the gut continue to increase. Both the bacteria and LPS enter the circulation, triggering an inflammatory response and exacerbating damage to vascular endothelial function, thereby contributing to the development of hypertension.

LPS and LTA are found in the cell walls of Gram-negative and Gram-positive bacteria. According to the results of this study, long-term airport noise exposure causes a significant increase in the concentrations of LPS and LTA, impairing the intestinal barrier function to a certain extent and thus allowing a large amount of LPS to pass into the circulation and cause an inflammatory reaction. Several recent studies have found that LPS and LTA can influence host epigenetic modifications and thus regulate inflammatory responses. By regulating these responses, they can cause DNA hypomethylation of several genes related to inflammatory pathways by inhibiting the expression of DNA methyltransferase (DNMT) and then increasing the expression of inflammatory factors [61,62,63,64]. Long-term exposure to airport noise can cause an inflammatory response in the colon, which can be manifested in severe damage to the morphological structure of the colon, which accords with the results of our HE staining experiments. These symptoms are evidence that long-term exposure to airport noise can cause morphological damage to the colon. Several studies have demonstrated that long-term noise exposure is followed by an increase in endotoxemic metabolism in the intestine, which may be correlated with long-term noise-induced inflammation, oxidative stress, metabolic disorders, and alterations in the intestinal flora [65,66].

## 5. Conclusions

In summary, long-term exposure to airport noise leads to increased blood pressure, an inflammatory response in colonic tissue, changes in the structural composition of the intestinal microbiota, and decreased levels of lower carboxylic acids. These changes lead to an increase in the concentration of the endotoxins LPS and LTA in the intestinal microbiota, which enter the circulation and stimulate the secretion of high levels of pro-inflammatory factors, thus increasing the degree of inflammation in the body. Additionally, decreased levels of TMA and TMAO lead to a disturbance in energy metabolism, which contributes to significant changes in the levels of other metabolites.

## Data Availability

The original contributions presented in this study are included in the article. Further inquiries can be directed to the corresponding authors.
